# Patterns and Environmental Drivers of Amphibian Alpha and Beta Diversity in the Huangshan Mountains, Southern Anhui, China

**DOI:** 10.1002/ece3.74556

**Published:** 2026-10-20

**Authors:** Suyue Wang, Ziyi Wang, Yujia Sun, Zhirong He, Qingyan Sun, Jiedong Wei, Yingxin Li, Meiting Liu, Jiayi Shi, Chunna Zhang, Siyu Wu, Yufeng Bai, Ziruo Zhang, Na Zhao, Supen Wang

**Affiliations:** ^1^ Anhui Provincial Key Laboratory of Biodiversity Conservation and Ecological Security in the Yangtze River Basin, College of Life Sciences Anhui Normal University Wuhu China; ^2^ Collaborative Innovation Center of Recovery and Reconstruction of Degraded Ecosystem in Wanjiang Basin Co‐Founded by Anhui Province and Ministry of Education, School of Ecology and Environment Anhui Normal University Wuhu China

**Keywords:** alpha and beta diversity, amphibian diversity, environmental drivers, Huangshan Mountains, human footprint, normalized difference vegetation index (NDVI), precipitation, species turnover

## Abstract

Understanding the spatial patterns of biodiversity and their environmental determinants is fundamental to ecology and conservation, particularly in mountainous transitional zones where species assemblages can shift rapidly. Amphibians, as highly sensitive organisms, are excellent indicators of environmental change, yet quantitative assessments of their diversity in the Huangshan Mountains—a biodiversity hotspot at the junction of the Palaearctic and Oriental realms in southern Anhui, China—remain limited. We conducted systematic line‐transect surveys across 200 grids (5 × 5 km) during the spring and autumn of 2023 and 2024, recording a total of 10,342 individuals belonging to 23 species, 20 genera, 9 families, and 2 orders. Three species ( *Fejervarya multistriata* , *Microhyla fissipes* , and *Bufo gargarizans* ) were identified as dominant, and two nationally protected species ( *Hoplobatrachus chinensis* and *Andrias davidianus* ) were detected. Inter‐annual alpha diversity was largely stable, with no significant differences in most metrics except species richness, but pronounced seasonal variation was observed, with spring supporting higher Shannon‐Wiener and Pielou evenness than autumn in both years. Using generalized additive models and model averaging, we found that annual precipitation and the normalized difference vegetation index (NDVI) were consistently the strongest positive predictors of Shannon‐Wiener diversity, Simpson diversity, and species richness, while species richness also declined significantly with increasing human footprint. Total beta diversity (Sørensen dissimilarity) was very high (0.981) and overwhelmingly driven by species turnover (97.86%) rather than nestedness. Partial Mantel tests and distance‐based redundancy analysis further revealed that environmental distances—particularly annual precipitation—significantly shaped overall beta diversity and its turnover component after accounting for geographic distance. These results highlight the predominant role of climatic and vegetation gradients in structuring amphibian assemblages in this subtropical mountainous region, providing baseline data to inform local conservation strategies.

## Graphical abstract

Based on systematic grid surveys across the Huangshan Mountains in southern Anhui, China, this study characterized amphibian alpha and beta diversity patterns and their environmental correlates. Annual precipitation and the normalized difference vegetation index (NDVI) consistently emerged as the primary positive drivers of both local diversity and community turnover, while the human footprint had a negative effect on species richness. Beta diversity was overwhelmingly dominated by species turnover, underscoring the importance of protecting the full gradient of precipitation and forested habitats to sustain regional amphibian assemblages.

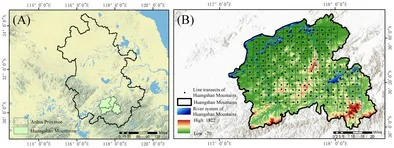

## 1 Introduction

Understanding the spatial distribution of biodiversity and the mechanisms that generate and maintain it remains a central pursuit in ecology and conservation biology (Edwards and Cerullo 2024; Wang et al. 2024). Species are not randomly distributed across landscapes; rather, their assemblages are shaped by a complex interplay of climatic conditions, topographic heterogeneity, habitat productivity, and anthropogenic pressures (Lawlor et al. 2024). Among vertebrates, amphibians are particularly sensitive to environmental variation owing to their permeable skin, physiological constraints, limited dispersal capacity, and dependence on both aquatic and terrestrial habitats (Riddell et al. 2026). These traits render them excellent biological indicators and ideal subjects for testing hypotheses about the environmental drivers of diversity patterns (Whitton et al. 2012). Because amphibian populations are declining worldwide due to habitat loss, climate change, pollution, and disease, spatially explicit assessments of their diversity have taken on renewed urgency (Luedtke et al. 2023).

Diversity can be examined through two complementary lenses (Tuomisto 2010). Alpha diversity captures local community structure in terms of species richness, evenness, and dominance, revealing how assemblages respond to site‐specific environmental conditions. Beta diversity, in contrast, quantifies the degree of compositional dissimilarity among communities across space or environmental gradients. Partitioning beta diversity into its turnover and nestedness components offers mechanistic insights: high turnover implies species replacement along gradients, often driven by environmental sorting or dispersal limitation, whereas strong nestedness suggests ordered species loss, frequently associated with extinction or habitat filtering (Baselga 2010). Disentangling these components is essential to understand whether conservation actions should aim to protect environmental heterogeneity or maintain key habitats that support more impoverished communities (Tscharntke et al. 2012).

Mountainous regions, with their steep climatic gradients and structural complexity, constitute natural laboratories for biodiversity research (Rahbek et al. 2019). The Huangshan Mountain Range in southern Anhui, China, exemplifies such a setting. Situated on the northern border of the mid‐subtropical zone, this area stands at the transitional zone between the Palaearctic and Oriental zoogeographical realms (Shi, He, et al. 2025). The interaction of a subtropical monsoon climate, rugged topography, and extensive subtropical evergreen broad‐leaved forests creates a mosaic of microhabitats that is expected to support diverse amphibian assemblages (Shi, Zhang, et al. 2025). Despite its biogeographic significance and recognized status as a biodiversity hotspot, quantitative, spatially replicated studies that simultaneously analyze amphibian alpha and beta diversity and their environmental correlates in this transitional landscape remain scarce.

Previous work in other systems has identified several key environmental determinants of amphibian diversity, including temperature, precipitation, vegetation productivity, proximity to water bodies, and intensity of human disturbance (Qian et al. 2007). However, the relative importance of these drivers can vary substantially among biogeographic regions and spatial scales, and collinearity among predictors often complicates statistical inference (Bini et al. 2009). In the Huangshan Mountains, fragmentary faunal inventories exist, but a comprehensive, grid‐based survey that controls for spatial autocorrelation and applies robust multivariate modeling to assess both alpha and beta diversity has not been conducted (Hong et al. 2025). This gap limits our ability to predict how amphibian communities in this transitional zone might respond to ongoing environmental change and to prioritize areas for conservation.

Here, we leverage systematically designed field surveys carried out during the peak activity seasons of 2023 and 2024 across 200 sampling grids in the Huangshan Mountains. Our objectives were threefold. First, we characterized amphibian species composition and alpha diversity patterns, examining both inter‐annual and seasonal variation. Second, we quantified beta diversity and partitioned it into turnover and nestedness components to infer the processes underlying community differentiation. Third, we evaluated the independent and combined effects of climatic, topographic, vegetation, and human‐disturbance variables on both alpha and beta diversity, using techniques that account for collinearity and spatial structure. Based on the region's strong precipitation and productivity gradients, we hypothesized that annual precipitation and the normalized difference vegetation index would be the primary positive drivers of alpha and beta diversity, that the human footprint index would exert a negative influence, and that species turnover would dominate beta diversity given the environmental heterogeneity characteristic of the study area. By providing a robust, spatially explicit baseline, this study aims to inform amphibian monitoring and conservation planning in the southern Anhui mountainous area.

## 2 Materials and Methods

### 2.1 Study Area

The study was conducted in the Huangshan Mountain Range, southern Anhui, China (29°59′–30°32′ N, 117°12′–118°21′ E), a biodiversity hotspot situated at the transition between the Palaearctic and Oriental zoogeographical realms (Liang 1998; Wang et al. 2022). The region has a subtropical monsoon climate with a mean annual temperature of 15°C–16°C and annual precipitation of 1400–2200 mm (Wang et al. 2021). The topography is dominated by mountains and hills, with the highest peak reaching 1884 m above sea level, creating a marked elevational gradient across the study area (Chen et al. 2023). The zonal vegetation is subtropical evergreen broad‐leaved forest (Wang et al. 2021). The study area covers approximately 5500 km^2^ across the districts of Huangshan, Guichi, Qingyang and Shitai.

### 2.2 Field Surveys

Amphibian surveys followed the grid‐based sampling framework of the national technical guidelines for county‐level amphibian and reptile diversity assessment. A total of 200 grids, each 5 × 5 km, were established across the study area (Therres et al. 2015). One line transect 200 m long and 2–4 m wide was placed in each grid, with a minimum distance of 5 km between adjacent transects (Cushman 2006; Rödel and Ernst 2004) (Figure 1). To maximize the probability of species detection, transects were deliberately positioned as close as possible to streams or ponds. This placement strategy was adopted because amphibians have highly permeable skin and depend on water bodies for reproduction, so their abundance and detection probability generally decline with increasing distance from water sources (Paudel et al. 2022). Surveys were conducted in spring (April–May) and autumn (September–October) of 2023 and 2024, corresponding to peak amphibian activity periods in this region (Liu et al. 2022; Pan et al. 2014). All surveys were carried out between 20:00 and 24:00 to coincide with the nocturnal activity of most amphibian species (Liu et al. 2022; Xiong et al. 2025). Survey effort was consistent across all four periods: all 200 transects were surveyed in each period, with positions held constant so that the same locations were sampled repeatedly, thereby maximizing detection probability and minimizing false absences. Each transect was walked at a speed of 2–3 km/h by a three‐person team (two observers and one recorder). Species were detected and identified by direct visual observation and acoustic identification of calls; individuals not reliably identifiable by these means were sampled non‐lethally and identified by DNA barcoding in the laboratory. For each encounter, we recorded the species, number of individuals, geographic coordinates and elevation (see Data S1 in Supporting Information). Species identification followed Fei et al. (2006, 2009) and Fei et al. (2012), with nomenclature updated according to Frost (2024). The national conservation status of recorded species was determined using the National Key Protected Wild Animals List (2023), and threat categories were assigned based on the Red List of China's Biodiversity (Jiang et al. 2021). To account for spatial autocorrelation, the geographic coordinates (Longitude and Latitude) of each site were included as spatial trend terms in the db‐RDA model. To verify that the model adequately controlled for spatial structure, we tested the model residuals for spatial autocorrelation using Moran's I, based on a row‐standardized spatial weights matrix defined by the five nearest neighbors (*k* = 5) of each site in projected coordinates (UTM Zone 50 N, EPSG: 32650).

**FIGURE 1:**
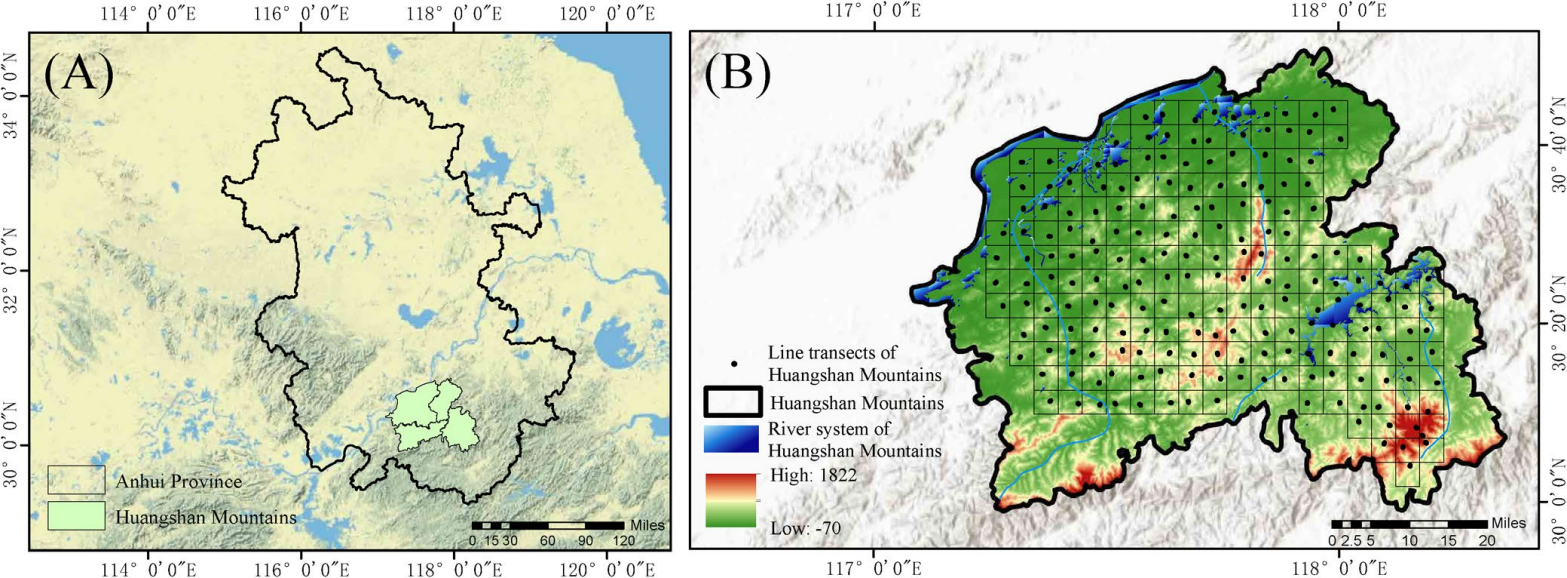
Location of the study area and distribution of amphibian survey transects in the Huangshan Mountains of southern Anhui Province. (A) Map of Anhui Province, highlighting the Huangshan Mountains in the southern part; (B) Topographic map of the Huangshan Mountains, indicating the 200 grids (5 km × 5 km) and the survey transects.

### 2.3 Environmental Variables

We considered climatic, topographic, biotic and human‐disturbance factors known to influence amphibian diversity (Duan et al. 2016; Ochoa‐Ochoa et al. 2012; Zancolli et al. 2014). Two climatic variables (annual precipitation and mean annual temperature), three topographic variables (elevation, aspect and slope), one biotic variables (the Normalized Difference Vegetation Index, NDVI),one landscape variable (Euclidean distance to the nearest major river) and one anthropogenic variable (the Human Footprint Index) were obtained from public repositories (http://www.resdc.cn/) (Liu et al. 2022; Xiong et al. 2025). These layers differed in their native spatial resolutions: annual precipitation and mean annual temperature had a resolution of 30 arc‐seconds (approximately 1 km); elevation, aspect, slope, distance to river and the Human Footprint Index had a resolution of 30 m; and NDVI had a resolution of 1 km. All environmental variables were extracted at the midpoints of the 200 sampling grids (5 × 5 km). Because the analysis was based on point extraction at grid midpoints rather than raster overlay or grid‐cell aggregation, no resampling was applied to unify the native resolutions across layers. This point‐based extraction approach avoids introducing artificial values through interpolation and preserves the original information of each data layer. All layers were projected to the WGS 1984 UTM Zone 48 N coordinate system and clipped to the Anhui provincial boundary in ArcGIS 10.8. Values of each variable were extracted for the midpoints of all 200 transects using the “Extract” tool.

### 2.4 Data Analysis

All statistical analyses were performed in R version 4.5.2 (R Core Team 2024).

*Alpha diversity and temporal variation*. For each transect, species richness (C), Shannon‐Wiener diversity index (H), Simpson diversity index (D), and Pielou evenness index (J) were computed. Dominant species were identified using the Berger‐Parker dominance index (Pi = Ni/N), with thresholds of Pi ≥ 0.1 (dominant), 0.01 < Pi < 0.1 (common), and Pi ≤ 0.01 (rare) (Shi, He, et al. 2025). Because the same transects were surveyed repeatedly across seasons and years, all temporal comparisons—both inter‐annual (2023 vs. 2024) and seasonal (spring vs. autumn within each year, and spring vs. autumn across both years combined)—were performed using Wilcoxon signed‐rank tests for paired samples (Wilcoxon 1945).

*Sampling completeness*. To assess whether the survey effort was sufficient to capture the regional species pool, total species richness was estimated using the Chao2 estimator, which accounts for species detected in only one or two samples (Chao 1987). The analysis was performed with the specpool function in the R package vegan version 2.7‐2 (Oksanen et al. 2025), using the presence/absence matrix of the 200 transects. Sampling completeness was calculated as the ratio of observed species richness to the Chao2 estimate.

*Environmental predictors*. To avoid collinearity, we calculated pairwise Spearman's rank correlations among all 10 initially extracted variables. When |ρ| > 0.8, one variable of each pair was removed, giving priority to factors with clearer biological interpretation for amphibians (Xiao et al. 2016). Six non‐collinear variables were retained: annual precipitation, NDVI, slope, aspect, distance to river, and the Human Footprint Index.

*Alpha diversity–environment relationships*. Spearman correlations were first computed between each alpha diversity metric and the six predictors. Variables showing significant correlations (*p* < 0.05) were then entered into generalized additive models (GAMs). Variance inflation factors (VIF) were checked, and variables were retained only if VIF < 5.0 (Zuur et al. 2009). Model selection was based on the corrected Akaike information criterion (AICc); when multiple models had ΔAICc < 2, model averaging was applied to the candidate set to obtain unconditional parameter estimates and variable importance weights (Burnham and Anderson 2004).

*Beta diversity and partitioning*. Pairwise Sørensen dissimilarity (βsor) was calculated from species presence/absence data. Total beta diversity was partitioned into turnover (βsim) and nestedness (βnes) components using the beta.pair function in the R package betapart (Baselga & biogeography 2010). The relative contribution of each component to total dissimilarity was expressed as a percentage.

*Drivers of beta diversity*. Geographic distances between transect midpoints were calculated with the distm function in the geosphere package, and environmental distances were computed as Euclidean distances on scaled environmental variables using vegdist in vegan. Partial Mantel tests (9999 permutations) were used to assess the relationship between βsor, βsim, and βnes and each environmental distance matrix while controlling for geographic distance. Distance‐based redundancy analysis (db‐RDA) was additionally performed to quantify the unique and shared contributions of the six environmental predictors to beta diversity patterns, using the capscale function in vegan.

## 3 Results

### 3.1 Survey Summary and Species Composition

Across the 200 transects, 10,342 amphibian individuals were recorded, belonging to 23 species, 20 genera, 9 families and 2 orders. Ranidae was the most speciose family (7 genera, 9 species, 39.13% of total richness), followed by Dicroglossidae (4 genera, 4 species, 17.39%). Microhylidae, Rhacophoridae and Salamandridae each contributed two species, whereas Bufonidae, Megophryidae, Hylidae and Cryptobranchidae were represented by a single species (Figure 2). Three species were dominant in the assemblage: *Fejervarya multistriata* (Pi = 0.43), *Microhyla fissipes* (Pi = 0.18) and *Bufo gargarizans* (Pi = 0.14). Two nationally protected Class‐II species, *Hoplobatrachus chinensis* and *Andrias davidianus* , were detected. To objectively assess sampling adequacy, we estimated total species richness using the Chao2 estimator. The Chao2 estimate was 23.995 (SE = 2.299; 95% CI = 23.069–37.273), corresponding to a sampling completeness of 95.85%. The species accumulation curves also approached an asymptote in both years (Figure 3). Overall, these results indicate that our sampling effort was sufficient to capture the majority of amphibian species in the study area.

**FIGURE 2:**
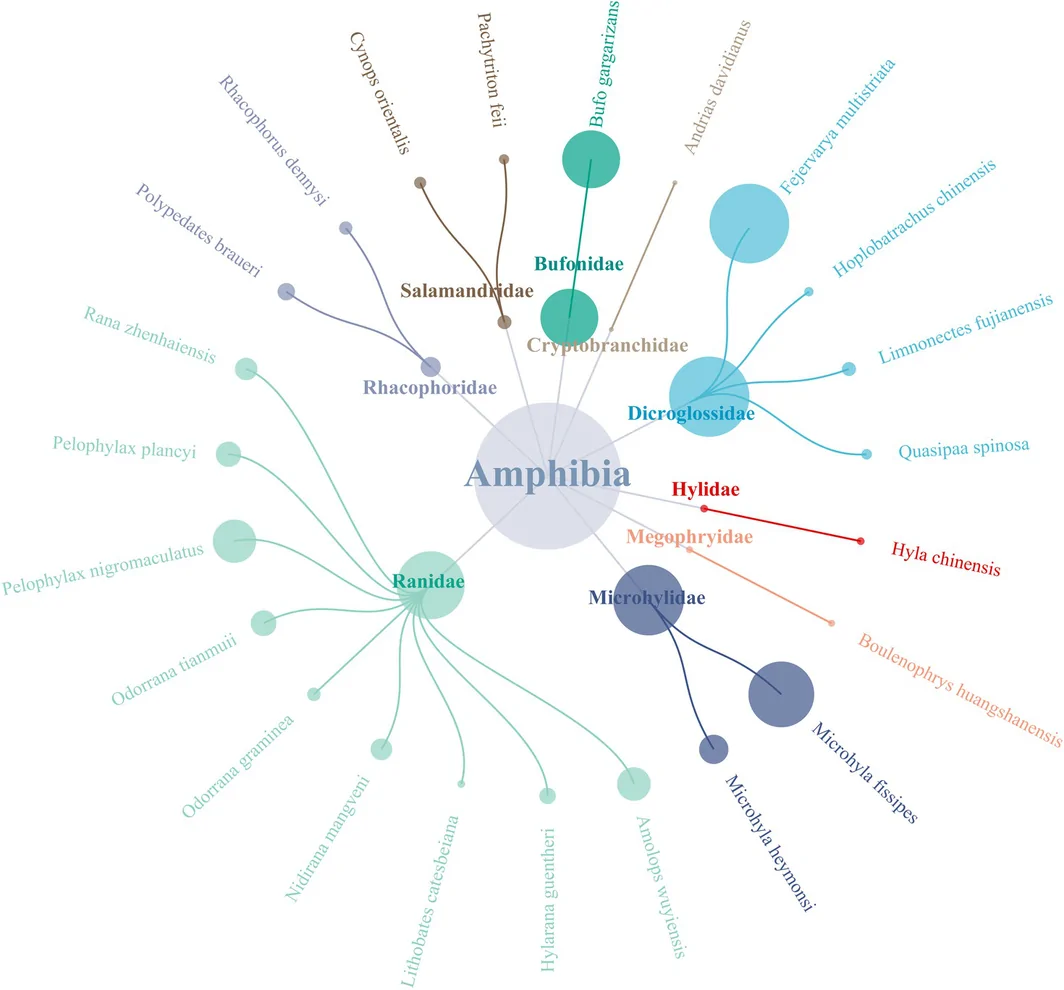
Species composition and relative abundance of amphibians in the Huangshan Mountains of southern Anhui Province. Circle size indicates relative abundance.

**FIGURE 3:**
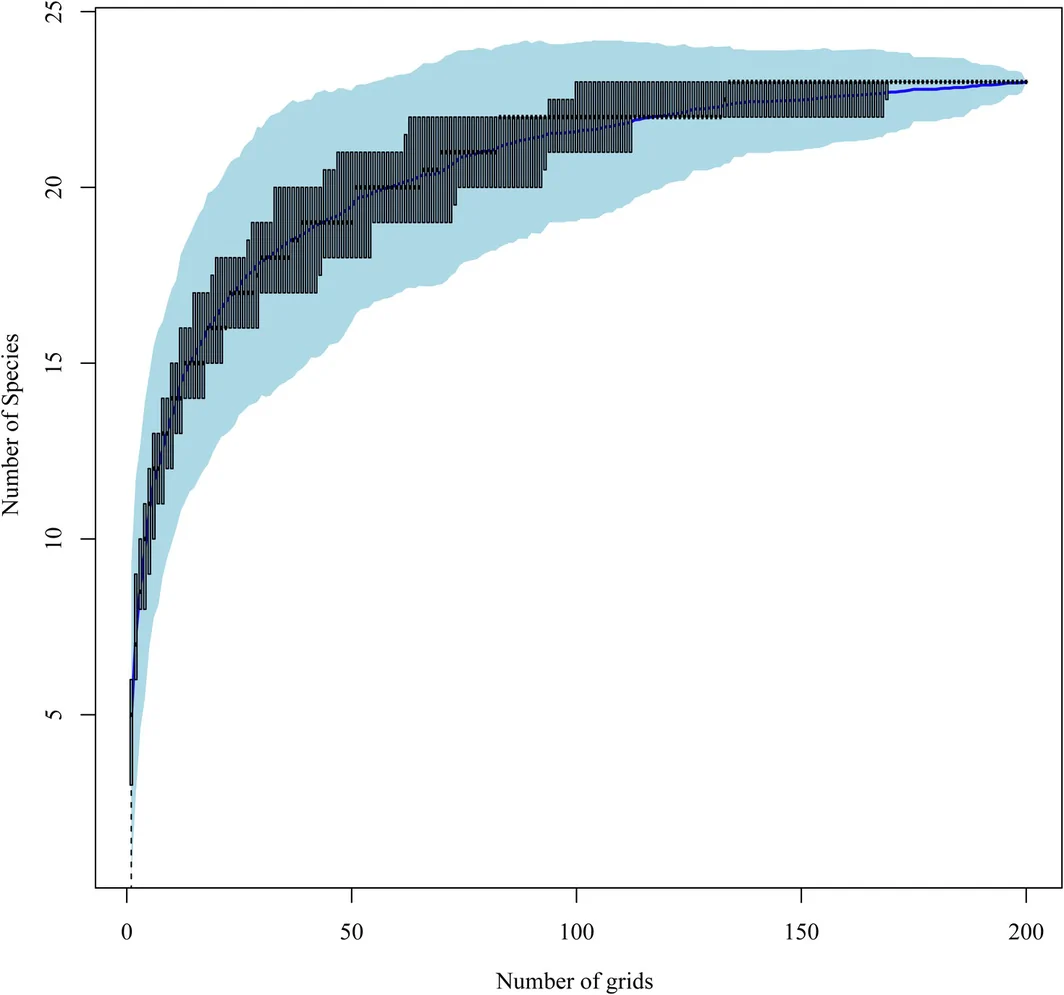
Species accumulation curves for amphibian surveys conducted in 2023 and 2024 in the Huangshan Mountains of southern Anhui Province.

### 3.2 Temporal Variation in Alpha Diversity

Inter‐annual variation in alpha diversity was generally weak, but not uniform across metrics. Wilcoxon signed‐rank tests showed no significant differences between 2023 and 2024 for Shannon‐Wiener diversity (V = 9140.5, *p* = 0.064), Simpson diversity (V = 8551.0, *p* = 0.562), Pielou evenness (V = 7241.5, *p* = 0.353), Berger‐Parker dominance (V = 8201.5, *p* = 0.833) or relative abundance (V = 8201.5, *p* = 0.833). However, species richness differed significantly between years (V = 2937.0, *p* < 0.001), although the median richness was identical (4 in both years), indicating that the difference arose from the distribution of paired differences rather than from a shift in the median. In contrast, pronounced seasonal variation was evident within each year. In 2023, Shannon‐Wiener diversity (V = 8920, *p* < 0.001), Simpson diversity (V = 8718, *p* = 0.035), Pielou evenness (V = 7955, *p* < 0.001), Berger‐Parker dominance (V = 5335, *p* = 0.026) and species richness (V = 6288, *p* < 0.001) were all significantly higher in spring than in autumn. In 2024, the same seasonal pattern was observed: Shannon‐Wiener diversity (V = 9441.5, *p* < 0.001), Simpson diversity (V = 9450.5, *p* = 0.031), Pielou evenness (V = 8879, *p* < 0.001), Berger‐Parker dominance (V = 6237.5, *p* = 0.039) and species richness (V = 6347, *p* < 0.001) were all significantly higher in spring than in autumn (Table 1). When data from both years were combined, the seasonal differences remained significant for Shannon‐Wiener diversity (V = 12,049, *p* < 0.001), Simpson diversity (V = 11,779, *p* < 0.001), Pielou evenness (V = 10,865, *p* < 0.001), Berger‐Parker dominance (V = 6266, *p* = 0.001) and relative abundance (V = 8444, *p* < 0.001), but not for species richness (V = 8188, *p* = 0.115). Overall, these results indicate that seasonal variation in alpha diversity was stronger and more consistent than inter‐annual variation, with species richness being the only metric that also differed significantly between years.

**TABLE 1:** Inter‐annual and seasonal differences in amphibian alpha diversity in the Huangshan Mountains of southern Anhui Province. Values are presented as median ± IQR. Differences were tested using Wilcoxon signed‐rank tests for paired samples.

Year	Alpha diversity index	Spring median (IQR)	Autumn median (IQR)	V	*p*
2023	Shannon‐Wiener diversity index	1.154 (0.637–1.561)	0.950 (0–1.332)	8920	*p* < 0.001
	Simpson diversity index	0.667 (0.480–0.752)	0.633 (0.444–0.741)	8718	*p* = 0.035
	Pielou evenness index	0.936 (0.792–0.994)	0.896 (0–0.946)	7955	*p* < 0.001
	Species richness	4.000 (2.000–5.000)	3.000 (1.000–4.000)	6288	*p* < 0.001
2024	Shannon‐Wiener diversity index	1.126 (0.687–1.581)	1.047 (0.375–1.386)	9441.5	*p* < 0.001
	Simpson diversity index	0.667 (0.500–0.778)	0.667 (0.500–0.781)	9450.5	*p* = 0.031
	Pielou evenness index	0.945 (0.817–0.994)	0.896 (0.541–0.946)	8879	*p* < 0.001
	Species richness	3.500 (2.000–5.000)	3.000 (1.750–5.000)	6347	*p* < 0.001

### 3.3 Alpha Diversity–Environment Relationships

Strong collinearity was found among the original environmental variables; for instance, elevation was highly correlated with mean annual temperature and annual precipitation, and slope with terrain ruggedness. After removing correlated variables, the six retained predictors were annual precipitation, NDVI, slope, aspect, distance to river, and the Human Footprint Index. Spearman correlations showed that Shannon‐Wiener diversity, Simpson diversity, and species richness were all significantly positively associated with annual precipitation, NDVI, slope, and distance to river, and significantly negatively associated with the Human Footprint Index. No significant correlation was detected between any alpha diversity metric and aspect (Figure 4).

**FIGURE 4:**
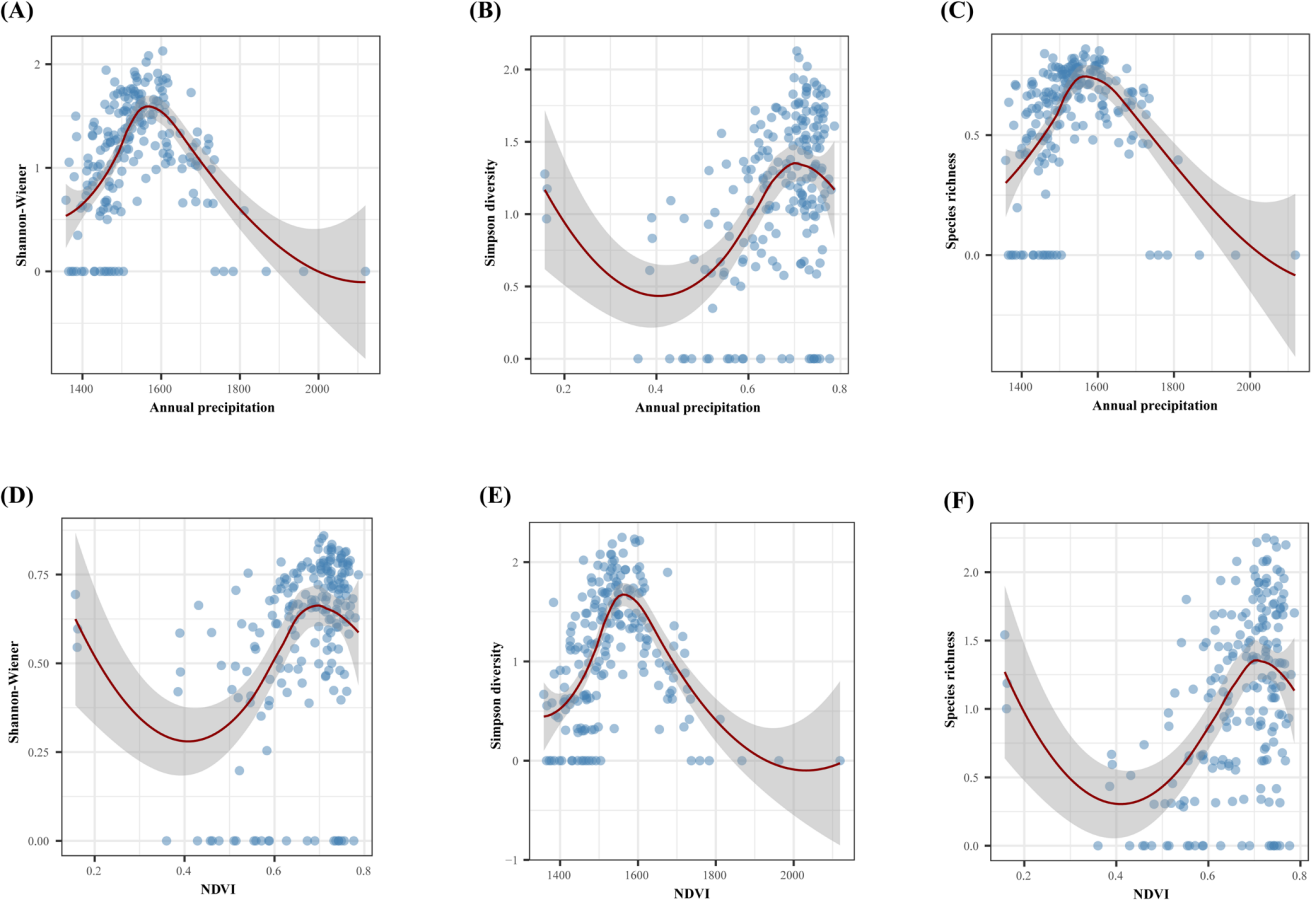
Effects of annual precipitation and NDVI on amphibian alpha diversity in the Huangshan Mountains of southern Anhui Province. (A), (B), and (C) show the relationships between annual precipitation and the Shannon‐Wiener diversity index, Simpson diversity index, and species richness, respectively. (D), (E), and (F) show the relationships between the Normalized Difference Vegetation Index (NDVI) and the Shannon‐Wiener diversity index, Simpson diversity index, and species richness, respectively. Blue dots represent observed values, red lines indicate the Spearman rank correlation fit, and shaded areas denote the 95% confidence intervals.

Generalized additive models and model averaging revealed that annual precipitation and NDVI were consistently the strongest predictors of alpha diversity (Table 2). Both variables were positively and significantly related to Shannon‐Wiener diversity, Simpson diversity, and species richness (all *p* < 0.001). In addition, species richness showed a significant negative relationship with the Human Footprint Index (*p* = 0.035), whereas this relationship was not significant for Shannon‐Wiener or Simpson indices. The model weights for annual precipitation and NDVI were 1.0 (or close to 1.0) for all three alpha diversity metrics, confirming their predominant role. Other variables, including slope and distance to river, had lower and non‐significant model‐averaged coefficients (Table 2).

**TABLE 2:** Model‐averaged model weights and *p*‐values for each alpha diversity index of amphibians in the Huangshan Mountains of southern Anhui Province.

Alpha diversity index	Environmental factors	Model weights	*p*
Shannon‐Wiener diversity index	Annual precipitation	1.000	< 0.001
	Normalized difference vegetation index	1.000	< 0.001
	Slope	0.312	0.253
	Distance to River	0.489	0.169
	Human Footprint Index	0.442	0.056
Simpson diversity index	Annual precipitation	1.000	< 0.001
	Normalized difference vegetation index	0.900	< 0.001
	Slope	0.530	0.164
	Distance to River	0.668	0.153
	Human Footprint Index	0.742	0.227
Species richness	Annual precipitation	1.000	< 0.001
	Normalized difference vegetation index	1.000	< 0.001
	Slope	0.500	0.370
	Distance to River	0.485	0.354
	Human Footprint Index	0.673	0.035

### 3.4 Beta Diversity Patterns and Drivers

Overall Sørensen dissimilarity among transects was 0.981, indicating strong compositional differentiation across the study area. Turnover (βsim = 0.960) accounted for 97.86% of total beta diversity, whereas nestedness (βnes = 0.021) contributed only 2.14%, demonstrating that community variation was overwhelmingly driven by species replacement rather than ordered species loss.

Distance‐based redundancy analysis (dbRDA) was used to quantify the explanatory power of environmental predictors for beta diversity, with geographic coordinates (Longitude and Latitude) included as spatial trend terms to account for spatial autocorrelation. The Moran's I test on the db‐RDA residuals was not significant (Moran's I = 0.0074, *p* = 0.771), indicating that the model residuals were spatially independent. The db‐RDA model was significant overall (F = 2.06, *p* = 0.0001; adjusted R^2^ = 4.1%), with Latitude (F = 4.91, *p* = 0.0001) and Bio12 (F = 6.61, *p* = 0.0001) as the only significant predictors. The marginal contributions of the other predictors were lower and non‐significant: Longitude (0.63%, *p* = 0.2059), NDVI (0.49%, *p* = 0.4139), River (0.71%, *p* = 0.1329), HFP (0.48%, *p* = 0.4202), Aspect (0.42%, *p* = 0.5617) and Slope (0.29%, *p* = 0.8355). Forward selection of dbRDA axes showed that only the first axis was significant (CAP1: F = 9.4219, *p* = 0.0001), while CAP2–CAP8 were not significant. These results identify annual precipitation and latitude as the key environmental and spatial gradients structuring amphibian beta diversity in the Huangshan Mountains, with species turnover being the dominant process.

## 4 Discussion

This study provides a spatially comprehensive assessment of amphibian diversity across the Huangshan Mountains, a biodiversity hotspot at the Palaearctic–Oriental transitional zone. Three main findings emerge: (i) amphibian alpha diversity was temporally stable between years but showed consistent seasonal variation, being higher in spring; (ii) annual precipitation and the normalized difference vegetation index (NDVI) were the primary environmental correlates of both alpha and beta diversity, while human footprint had a moderate negative effect on species richness; and (iii) amphibian community dissimilarity was exceptionally high and overwhelmingly driven by species turnover, not nestedness. These patterns align with the view that in topographically complex and climatically heterogeneous regions, species replacement along environmental gradients, rather than ordered species loss, structures communities.

The observed seasonal shift in alpha diversity is ecologically intuitive (Tonkin et al. 2017). Spring surveys coincide with the peak breeding period for many amphibian species in subtropical China, when adults congregate at breeding sites and are more detectable; autumn surveys capture the post‐breeding dispersal phase, often with juveniles dominating counts (Fe et al. 2010). The higher Shannon‐Wiener and Pielou evenness values in spring therefore likely reflect both higher activity and a more equitable distribution of individuals among species during reproduction, whereas in autumn some species (e.g., *F. multistriata* ) may become numerically overwhelming, lowering evenness and Simpson diversity (D = 1 − Σ(pᵢ)^2^). Most alpha diversity metrics did not differ significantly between years, indicating that the amphibian assemblages were largely stable over the two‐year period, which also supports the reliability of the data used for the spatial analyses in this study. However, species richness did differ significantly between years, suggesting that the number of species detected per transect varied more than expected by chance, even though the median richness remained unchanged. A longer time series is therefore required to robustly assess interannual stability, and even longer‐term monitoring data are still needed to identify directional changes in the community.

Among the environmental factors examined, annual precipitation emerged as the most robust predictor of both alpha and beta diversity. This finding is consistent with numerous studies that identify water availability as a primary constraint on amphibian distributions (Buckley and Jetz 2007; Cunningham et al. 2016; Liao et al. 2025; Winter et al. 2016; Zhao et al. 2022). In the Huangshan Mountains, precipitation likely influences breeding‐site hydroperiod, ambient humidity and the moisture of terrestrial microhabitats, all of which are critical for amphibian survival and reproduction. The positive association between NDVI and all alpha diversity metrics, and its significant link to beta diversity turnover, can be interpreted as the effect of vegetation productivity and structural complexity: dense, evergreen broad‐leaved forests provide cooler, wetter microclimates and abundant invertebrate prey, supporting richer and more stable amphibian assemblages. Importantly, both precipitation and NDVI remained highly significant in multivariate models after accounting for other variables, and model‐averaging gave them full or near‐full model weights, underscoring their independent influence.

The Human Footprint Index, which integrates population density, land use and infrastructure, was negatively correlated with species richness, a pattern that corroborates the detrimental effects of human disturbance on amphibian communities (Venter et al. 2016). However, the relationship was significant only for species richness and not for Shannon‐Wiener or Simpson indices. This may indicate that moderate disturbance can simplify communities by filtering out sensitive species without drastically altering overall diversity if common, disturbance‐tolerant species (such as *F. multistriata* ) remain abundant. The lack of a significant effect on evenness and dominance suggests that human footprint mainly acts by eliminating rare or specialized species, reducing richness, while the remaining dominant species maintain overall abundance patterns (Cazalis 2022; Hillebrand et al. 2008). No significant effect of slope, aspect, or distance to river was found in the ultimate models, implying that in this landscape, climatic and vegetation gradients may override local topographic and hydrographic factors at the grain of our analysis. However, these variables did show significant simple correlations with alpha diversity, so their influence is likely expressed through collinearity with precipitation and NDVI.

The beta diversity analysis revealed a striking predominance of the turnover component, accounting for nearly 98% of total dissimilarity. This result strongly suggests that amphibian communities are structured by environmental sorting across the strong precipitation and productivity gradients of the study area, rather than by dispersal limitation leading to nested species loss (Cunningham et al. 2016; Riddell et al. 2026). Such a pattern is expected in mountainous regions with high environmental heterogeneity (Hong et al. 2025). Distance‐based redundancy analysis (dbRDA) showed that the overall model was significant (F = 2.0642, *p* = 0.0001; adjusted R^2^ = 4.1%), and that annual precipitation (F = 6.6053, *p* = 0.0001) and latitude (F = 4.9122, *p* = 0.0001) were the only significant predictors of beta diversity, together accounting for the largest marginal contributions to the total sum of squares (Bio12: 1.797; Latitude: 1.337). In contrast, longitude, NDVI, slope, aspect, distance to river and the Human Footprint Index had non‐significant marginal effects after controlling for the other predictors (all *p* > 0.13). Forward selection of dbRDA axes further showed that only the first axis was significant (CAP1: F = 9.4219, *p* = 0.0001), whereas CAP2–CAP8 were not significant. Moreover, the Moran's I test on the model residuals was not significant (Moran's I = 0.0074, *p* = 0.771), indicating that the residuals were spatially independent and that the inclusion of geographic coordinates as spatial trend terms adequately controlled for spatial autocorrelation. These results indicate that annual precipitation, together with a broad latitudinal gradient, is the key environmental driver structuring amphibian beta diversity in the Huangshan Mountains, with species turnover being the dominant process. The fact that turnover, not nestedness, drove the pattern implies that conservation efforts aimed at maintaining the full regional species pool should prioritize protecting the full range of environmental conditions—particularly the wetter, more‐vegetated high‐precipitation zones—rather than focusing only on a few species‐rich sites (Wang et al. 2024).

Several limitations should be acknowledged. First, although the 2 years of surveys captured overall species richness well, as indicated by the Chao2 estimate (sampling completeness = 95.85%), the temporal scope is insufficient to assess trends under climate change. Second, surveys were conducted exclusively in spring and autumn, and no summer surveys were carried out. Summer is the peak breeding season for many amphibian species in subtropical China, when breeding choruses and larval stages are most conspicuous. The absence of summer surveys may therefore have reduced our ability to detect species with peak activity or reproduction in summer and may have underestimated the full seasonal diversity of the assemblage. Future studies should include summer surveys to cover the complete annual activity cycle of amphibians in this region. Third, surveys were conducted exclusively at night, which may have reduced detection of species with diurnal or crepuscular activity in the surveyed season. Fourth, environmental variables were extracted at a 5‐km grid resolution, which may mask fine‐scale habitat features important for amphibians, such as the availability of temporary ponds or coarse woody debris. Fifth, the sampling design employed only one 200‐m transect per 5 × 5 km grid, which, although logistically feasible across a large mountainous region, represents a relatively low sampling intensity and may not fully capture local heterogeneity in amphibian assemblages. Although transects were deliberately positioned near streams or ponds to maximize detection probability, the limited spatial replication within each grid may reduce the ability to detect fine‐scale species–environment relationships. We therefore acknowledge that future studies should consider increasing sampling intensity and spatial replication—for example, by establishing multiple transects per grid or by adopting a hierarchical sampling design—to better capture local‐scale variation in amphibian diversity and to strengthen the robustness of spatial inference. Future work could incorporate such microhabitat data and potentially use acoustic monitoring to enhance detection, particularly during the summer breeding season. Nevertheless, the strong and ecologically coherent relationships detected here provide a solid baseline.

In conclusion, this study demonstrates that amphibian diversity in the Huangshan Mountains is primarily structured by climatic moisture and vegetation productivity, with human disturbance playing a secondary role mainly via species loss. The dominance of species turnover in beta diversity highlights the conservation value of environmental gradients in this transitional mountain ecosystem. Protecting the remaining high‐precipitation, forested areas from further human encroachment would be a priority action for safeguarding the region's amphibian assemblages.

## Author Contributions

**Suyue Wang:** formal analysis (lead), investigation (lead), writing – original draft (lead). **Ziyi Wang:** investigation (supporting). **Yujia Sun:** investigation (supporting). **Zhirong He:** investigation (supporting). **Qingyan Sun:** investigation (supporting). **Jiedong Wei:** investigation (supporting). **Yingxin Li:** investigation (supporting). **Meiting Liu:** investigation (supporting). **Jiayi Shi:** investigation (supporting). **Chunna Zhang:** investigation (supporting). **Siyu Wu:** investigation (supporting). **Yufeng Bai:** investigation (supporting). **Ziruo Zhang:** investigation (supporting). **Na Zhao:** investigation (supporting). **Supen Wang:** conceptualization (lead), formal analysis (lead), writing – original draft (supporting).

## Funding

This work was supported by Amphibian and Reptile Survey Project in the Jianghuai Hilly Region and Huaihuai Plain of Anhui Province, 2025BFAFZ02069‐2.

## Ethics Statement

All animal experimental procedures received ethics review and approval from Anhui Normal University (AHNU‐ET2022014).

## Conflicts of Interest

The authors declare no conflicts of interest.

## Supporting information


**Data S1:** Supporting Information.

## Data Availability

All datasets supporting the current study's findings are provided within Data S1 in the Supporting Information.
